# Novel *TTG1* Mutants Modify Root-Hair Pattern Formation in Arabidopsis

**DOI:** 10.3389/fpls.2020.00383

**Published:** 2020-04-07

**Authors:** Yun Long, John Schiefelbein

**Affiliations:** ^1^Maize Research Institute, Sichuan Agricultural University, Chengdu, China; ^2^Department of Molecular, Cellular, and Developmental Biology, University of Michigan, Ann Arbor, MI, United States

**Keywords:** cell differentiation, cell fate, pattern formation, root development, root hair, transcriptional network

## Abstract

The patterning of root-hair and non-hair epidermal cells in the Arabidopsis root is governed by a network of transcriptional regulators. The central MYB-bHLH-WD40 (MBW) transcriptional complex includes the WD40-repeat protein TRANSPARENT TESTA GLABRA1 (TTG1). To clarify the role of TTG1, we describe the identification and analysis of two new *ttg1* mutants. Each of these mutants contains a single nucleotide change in the *TTG1* gene, which causes a single amino-acid substitution in the predicted TTG1 protein and alters root-hair pattern formation. Surprisingly, these new *ttg1* mutants exhibit decreased root-hair formation, particularly in the *caprice (cpc)* mutant background, rather than increased root-hair formation as reported for strong *ttg1* mutants. We show that the unique phenotype of these mutants is due to differential effects of the altered TTG1 proteins on target gene expression, associated with a weakened ability to interact with its GLABRA3 bHLH partner. These findings demonstrate the crucial role of TTG1 for the appropriate balance of target gene activation to achieve the proper pattern of epidermal cell types during Arabidopsis root development.

## Introduction

The development of the Arabidopsis root epidermis has been widely used as a simple model for understanding cell-type specification and pattern formation in plants (Duckett et al., 1994; Schiefelbein et al., 2009). There are two distinct cell types produced in the root epidermis, root-hair cells and non-hair cells, and these are arranged in a position-dependent pattern (Grebe, 2012). Developing epidermal cells located between two underlying cortical cells (the “H” cell position) are specified as root-hair cells, and epidermal cells located over a single cortical cell (the “N” cell position) are specified as non-hair cells (Cormack, 1962; Dolan and Roberts, 1995; Berger et al., 1998). Over the past 20 years, a large collection of studies have uncovered a highly orchestrated network of transcriptional regulators responsible for establishing position-dependent gene expression leading to the two cell fates (Bruex et al., 2012; Schiefelbein et al., 2014). The basic component of this network is a central complex containing a WD40-repeat protein encoded by *TRANSPARENT TESTA GLABRA 1* (*TTG1***) (**Galway et al., 1994; Walker et al., 1999), bHLH proteins encoded by the functionally redundant *GLABRA3* and *ENHANCER OF GLABRA3* (*GL3/EGL3*) (Payne et al., 2000; Bernhardt et al., 2003, 2005), and an R2R3-type MYB protein encoded by *WEREWOLF* (*WER*) (Lee and Schiefelbein, 1999). This MYB-bHLH-WD40 (MBW) complex preferentially accumulates in non-hair cells where it directly promotes transcription of *GLABRA 2* (*GL2*) (Di Cristina et al., 1996; Masucci et al., 1996). *GL2* encodes an HD-ZIP transcription factor that activates transcription of non-hair cell differentiation genes and inhibits the expression of root-hair promoting genes such as *ROOT HAIR DEFECTIVE 6* (*RHD6***) (**Masucci and Schiefelbein, 1994; Yi et al., 2010). As a result, null mutations of *WER*, *GL3/EGL3*, and *TTG1*, or *GL2* cause a lack of non-hair cells and exhibit a hairy root phenotype.

The MBW complex also mediates lateral inhibition by promoting transcription of the single-repeat R3-type MYB genes *CAPRICE* (*CPC*), *TRIPTYCHON* (*TRY*), and *ENHANCER OF TRY AND CPC 1* (*ETC1*) preferentially in the N-position cells (Wada et al., 1997; Schellmann et al., 2002; Kirik et al., 2004; Simon et al., 2007). The resulting proteins then appear to move through plasmodesmata to accumulate in the adjacent H-position cells where they compete with WER for binding to GL3/EGL3 bHLHs and generate a non-functional complex (Lee and Schiefelbein, 2002; Kurata et al., 2005; Song et al., 2011). As a result, the H-position cells express *GL2* at a relatively low level and *RHD6* at a relatively high level, which specifies the root-hair cell fate. The CPC, TRY, and ETC1 proteins are functionally redundant, although CPC is most abundant and plays the major role in root-hair patterning (Simon et al., 2007). Null mutations of *CPC, TRY*, and/or *ETC1* produce less root-hair cells and more non-hair cells. In addition to the movement of CPC/TRY/ETC1 from N cells to H cells, the *GL3*/*EGL3* transcripts are found to preferentially accumulate in the H cells rather than the N cells, due to the negative transcriptional regulation of these genes by the MBW complex (Bernhardt et al., 2005; Kang et al., 2013). The GL3/EGL3 proteins are translocated from the H cells to the N cells. The opposite movement of CPC/TRY/ETC1 and GL3/EGL3 forms an intercellular mutual reinforcing loop and provides a robust root-hair patterning system (Schiefelbein et al., 2014). The patterning system is initiated by position cue(s) acting through the SCRAMBLED (SCM) LRR receptor-like kinase to influence the relative abundance of the MBW complex in the H and N positions (Kwak et al., 2005; Kwak and Schiefelbein, 2007, 2008).

In addition to root-hair patterning, the *TTG1* gene is involved in several other developmental and biochemical pathways in Arabidopsis including trichome patterning, anthocyanin accumulation, seed coat pigmentation, and seed mucilage production (Walker et al., 1999; Miller et al., 2016). For each pathway, *TTG1* appears to participate via a MBW complex, although different MYB and bHLH components are used for the five pathways (Zhang and Schrader, 2017). In trichome patterning, GLABROUS1 (GL1) is functionally equivalent to WER and forms a GL1-GL3/EGL3-TTG1 complex to promote trichome differentiation (Larkin et al., 1999; Kirik et al., 2005). Interestingly, the TTG1 exhibits an opposite effect on trichome formation when compared to its effect on root-hair formation. The *ttg1* mutants produce no trichomes (a glabrous phenotype), but it produces excess root hairs (a hairy root phenotype) (Galway et al., 1994; Larkin et al., 1999). The proanthocyanidin biosynthetic pathway is blocked in *ttg1* mutants causing a transparent testa phenotype in the seed coat (Haughn and Chaudhury, 2005; Gonzalez et al., 2009). In wild type seedlings, purple anthocyanin pigments are present in the hypocotyl, while *ttg1* mutants completely lack the accumulation (Baudry et al., 2004; Gonzalez et al., 2008). The *TTG1* gene encodes a protein of 341 amino acid residues with four WD40 repeats (Walker et al., 1999), and many *ttg1* alleles have been identified, such as *ttg1-1* (Q317stop) (Koornneef, 1981), *ttg1-9* (S282F) (Larkin et al., 1994b), *ttg1-10* (G→A in 5’UTR) (Larkin et al., 1994a), and *ttg1-13* (deletion) (Larkin et al., 1999). These mutant lines exhibit pleiotropic phenotypes, including excessive (ectopic) root hairs, glabrous leaves, and yellow seed coat, although the severity of the mutant alleles varies (Bouyer et al., 2008; Zhang and Schrader, 2017).

To further investigate the cell fate determination mechanism in the Arabidopsis root epidermis, we sought to identify new genes or alleles that control the cell-type specification process. In this study, two novel mutant alleles of *TTG1*, designated *ttg1-23* and *ttg1-24*, were identified from an enhancer screen in the *cpc-1* mutant background. Both of these mutant *ttg1* alleles were found to contain a single nucleotide change causing a single-residue substitution in the TTG1 protein and generating a novel root epidermis phenotype. The detailed analysis of these mutants provide new insights into the function of TTG1 and its role in root-hair cell patterning.

## Materials and Methods

### Plant Materials and Growth Conditions

The following mutant and transgenic lines have been previously described: *cpc-1* (Wada et al., 1997), *ttg1-1* (Koornneef, 1981), *ttg1-13* (Larkin et al., 1999), *ttg1-9* (Galway et al., 1994), and *GL2:GUS* (Masucci et al., 1996). Seeds were surface sterilized with 30% bleach and 0.02% Triton-X100 and sown on mineral nutrient mix media solidified with 0.3% Gelrite as described (Schiefelbein and Somerville, 1990). Four-day-old seedlings incubated vertically at 23°C under continuous light were used for all experiments. For plant propagation, 10-day-old seedlings were transplanted to soil and grown in growth chambers under long-day light cycle at 23°C (16 h day) and 18°C (8 h night).

### Genetic Screening, Positional Mapping, and Whole Genome Sequencing

Seed mutagenesis of the *cpc-1 GL2:GUS* line (Wassilewskija [Ws] ecotype) with ethyl methanesulfonate (EMS) was performed as previously described (Estelle and Somerville, 1987). The *cpc-1 ttg1-23* and *cpc-1 ttg1-24* mutants were identified from the M_2_ population by visually screening for reduced root hair density using a dissection microscope. The F_2_ and F_3_ offspring from a cross between *cpc-1 ttg1-23* and a Columbia wild-type plant were analyzed using multiple simple sequence length polymorphism (SSLP) markers (Bell and Ecker, 1994), and strong linkage was identified with marker NGA139 (position 8.4 Mb on chromosome 5). At the same time, a pool of 122 individuals of F_3_ offspring together with *cpc-1* and Columbia were collected for whole genome sequencing. The genomic DNA was prepared with DNeasy Plant Maxi Kit (Qiagen) and samples were sequenced on an Illumina HiSeq 4000 platform in a 150-bp paired end run. The alignment of reads and variants calling were performed with MiModD 0.1.9.

Genotyping of *ttg1-23* and *ttg1-24* was performed with the Derived Cleaved Amplified Polymorphic Sequencing (dCAPS) (Neff et al., 2002) technique, using primers listed in Supplementary Table S1.

### Transgene Construction and Plant Transformation

The *TTG1* genomic DNA including 3-kb 5’ promotor sequence, 1-kb gene sequence and 1-kb 3’ terminal sequence was cloned using Phusion (NEB) and integrated into the pCB302 binary vector (digested with *Xba*I and *Bam*HI) (Xiang et al., 1999) using the HiFi assembly system (NEB). Cloning primers are listed in Supplementary Table S1. The verified *TTG1:TTG1* construct was then transformed into *ttg1-23*, *ttg1-24*, *cpc-1 ttg1-23*, and *cpc-1 ttg1-24* mutant plants through floral dipping as previously described (Clough and Bent, 1998).

To construct the *TTG1:TTG1-EYFP* transgene, the *TTG1* genomic segment including 3-kb 5’ promotor sequence, 1-kb stop codon deleted coding sequence, 1-kb 3’ terminal sequence and EYFP fragment were combined using Phusion (NEB) and integrated into the pCB302 binary vector (digested with *Xba*I and *Bam*HI) using the HiFi assembly system (NEB). The *TTG1:TTG1(23)-EYFP* and *TTG1:TTG1(24)-EYFP* transgenic constructs were created in the same manner, but the wild-type coding sequence was replaced with the *ttg1-23* and *ttg1-24* mutants, respectively. Cloning primers are listed in Supplementary Table S1. Sequence-verified *TTG1:TTG1-EYFP*, *TTG1:TTG1(23)-EYFP*, and *TTG1:TTG1(24)-EYFP* constructs were then transformed into *ttg1-13* mutant plants through the floral dipping method. After plant transformation, T_0_ plants were grown and T_1_ seeds were harvested and subjected to glufosinate-ammonium (PESTANAL^®^, Sigma-Aldrich) selection. The resistant T_1_ seedlings were grown and selfed to obtain T_2_ seeds, and single insertion lines were identified by analyzing the segregation of individual T_2_ populations for their chemical resistance and root-hair pattern. From each transformation experiment, homozygous T3 populations from at least three independent single-insertion lines were identified and used for further experiments.

### Microscopy and Image Analysis

The quantification of root epidermal cell types was performed using a bright field compound microscope, following brief staining with toluidine blue as described (Wang et al., 2019). Cell positions were determined by inspecting the underlying cortical cells, and hair cells were defined by the presence of a visible protrusion regardless of its length. For each genotype, three independent biological replicates were performed. For each replicate, a total of 10 seedlings were analyzed and 10 cells were scored in both the H and N positions in each seedling (total of 100 cells).

Histochemical analysis of GUS fusion reporter lines was performed essentially as previously described (Masucci et al., 1996; Wang et al., 2019). Specifically, 10 μl/mL of X-Gluc (Gold Biotechnology) substrate was used for *GL2:GUS* seedling incubation (20 min at 37°C). To generate histograms representing the *GL2:GUS* signal distribution, wild-type, *ttg1-23* and *ttg1-24* roots were stained and photographed under the same conditions. For each root, 10 continuous cells in one file were analyzed from the oldest cell prior to rapid elongation (i.e., the cell’s length exceeds its width) and continuing toward the root tip. For all cells, the GUS signal was measured using the same region of interest frame and the mean values were plotted onto histograms. For each genotype, three independent biological replicates were performed.

Fluorescence imaging was performed using a TCS SP5 DM6000B broadband confocal microscope (Leica) with 20× dry lens. Seedlings roots were briefly stained in propidium iodide (PI) for cell wall visualization. Default excitement and emission settings for YFP and PI signals were used for imaging. Care was taken to ensure each root was imaged on similar *Z*-axis positions marked by the maximum nucleus size.

### Pigment Accumulation and Trichome Analysis

Photographs of seed coat color were taken with a Canon EOS Rebel XSi. The hypocotyls of three-day-old seedlings grown on mineral nutrient mix media were examined and photographed for their anthocyanin accumulation using a Wild Makroskop M420 photomacroscope. Trichomes from one of the first two postembryonic leaves (referred to as the “first true leaves”) were analyzed using a Leica MS5 stereomicroscope (Larkin et al., 1999). Only trichomes that clearly protruded from the surface of the leaf were counted. For each genotype, three independent biological replicates were performed. For each replicate, trichomes were counted on one of the first true leaves from 10 independent plants. A trichome cluster was defined as two or more trichomes located immediately adjacent to each other, without intervening adjacent cells, and these were analyzed as described (Larkin et al., 1994a).

### RNA Extraction and Reverse Transcriptase Quantitative PCR (RT-qPCR)

Root tips including the meristematic zone, elongation zone, and early maturation zone were used for total RNA extraction with the RNeasy Plant Mini kit (QIAGEN) (Huang and Schiefelbein, 2015). RNA was treated with the RQ1 DNase (Promega), and cDNA was synthesized using the SuperScript First-Strand Synthesis System (Invitrogen). The qPCR experiments used the Radiant Green Hi-Rox qPCR Kit (Alkali Scientific Inc.) and was conducted with the StepOnePlus real-time PCR system (Applied Biosystems). The relative transcript amounts were determined using the Delta-Delta-Ct method (Livak and Schmittgen, 2001). The *GAPCP2* gene (*AT1G16300*, encoding a GAPDH isoform) was used as the internal reference gene. For each genotype, three independent biological replicates were performed. Primers used for RT-qPCR are listed in Supplementary Table S1.

### Yeast Two-Hybrid Assays

Yeast two-hybrid assays were conducted as previously described (Lee and Schiefelbein, 1999; Bernhardt et al., 2003). The entire coding region of the GL3 cDNA was joined as a C-terminal fusion to the yeast GAL4 DNA-binding domain in pGBT9 to generate the in-frame protein fusion BD-GL3. The GAL4 transcriptional activation domain in pGAD424 was fused to the full-length TTG1-coding region of wild-type, *ttg1-23* and *ttg1-24* to generate AD-TTG1(WT), AD-TTG1(*ttg1-23*), and AD-TTG1(*ttg1-24*), respectively. After transformation into yeast strain AH109, the β-galactosidase assays were performed on at least three individual transformants (3 biological replicates) for each combination of constructs using the Yeast β-Galactosidase Assays Kit (Thermo Fisher Scientific).

## Results

### Isolation of a Novel Root Epidermis Development Mutant

**FIGURE 1:**
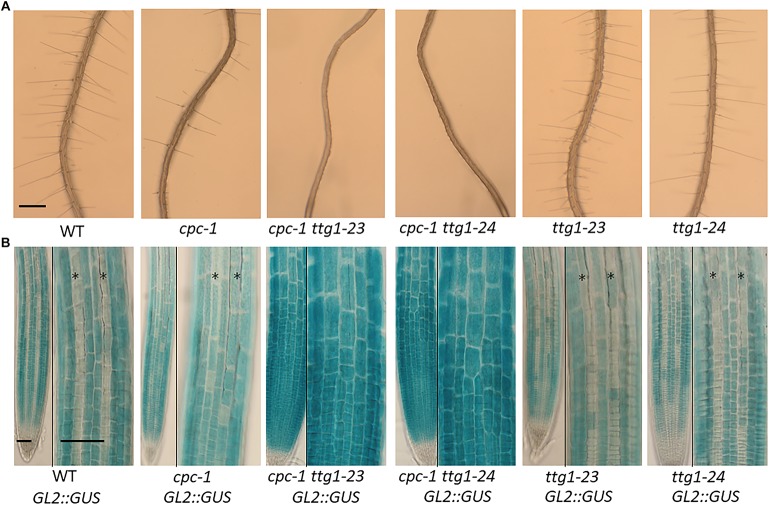
Characterization of the *ttg1-23* and *ttg1-24* mutants. **(A)** Seedling roots of wild type, *cpc-1*, *cpc-1 23*, *cpc-1 24*, *23*, and *24* displaying their root hair phenotypes. Bar = 200 μm. **(B)** *GL2:GUS* reporter expression in seedling root tips of wild type, *cpc-1*, *cpc-1 23*, *cpc-1 24*, *23*, and *24*. Stars indicate H-position epidermal cell files. For each panel, the left and right images show the same root under different magnification. Bar = 50 μm.

**FIGURE 2:**
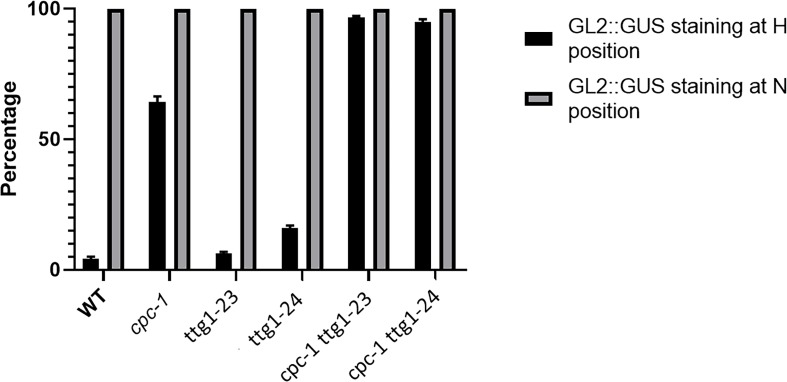
Histograms of *GL2:GUS* expression in H-position and N-position root epidermal cells of WT, *cpc-1*, *ttg1-23*, *ttg1-24*, *cpc-1 ttg1-23*, and *cpc-1 ttg1-24* seedling root tips. Ten cells from each of the H and N positions in each root were measured. Ten seedling roots were analyzed for each replicate. Three independent biological replicates were performed for each genotype. Data shown are means (+SD).

The *cpc-1* mutant produces approximately 40% of the number of root hairs found in wild-type roots (Lee and Schiefelbein, 2002), which provides a perturbed root-hair pattern useful for secondary genetic screens to discover new root epidermis development genes. We performed an ethyl methanesulfonate (EMS)-based enhancer screen in the *cpc-1 GL2:GUS* background and identified seedlings in subsequent generations exhibiting a more extreme reduced root-hair phenotype. One of the resulting lines, ultimately designated as *cpc-1 ttg1-23* (see below), generated very few root hairs (approximately 4% of the wild type number; Figure 1A and Table 1) and exhibited increased ectopic expression of *GL2:GUS* in differentiating H-position cells as compared to *cpc-1* (Figures 1B, 2). Further, we observed a 3:1 segregation ratio of the *cpc*-like phenotype to the double mutant-like phenotype (hairless) among offspring from a self-pollinated *cpc-1/cpc-1 ttg1-23/*+ population, indicating that the *ttg1-23* mutation is recessive. These results indicate that the new *cpc-1* enhancing mutation acts at an early developmental stage (upstream of *GL2*) to cause a change in root epidermal cell fate (from root-hair cells to non-hair cells).

**TABLE 1:** Specification of cell types in the root epidermis^1^.

	H cell position		N cell position	
	Hair	Non-hair	Hair	Non-hair
Genotype	cells (%)	cells (%)	cells (%)	cells (%)
Wild type (WS)	95.2 ± 0.8	4.8 ± 0.8	0.2 ± 0.4	99.8 ± 0.4
*cpc-1*	36.0 ± 2.2	64.0 ± 2.2	0.0 ± 0.0	100.0 ± 0.0
*23*	93.5 ± 2.1	6.5 ± 2.1	0.7 ± 1.2	99.3 ± 1.2
*24*	84.3 ± 3.8	15.3 ± 3.8	1.2 ± 1.8	98.8 ± 1.8
*cpc-1 23*	4.2 ± 0.8	95.8 ± 0.8	0.0 ± 0.0	100.0 ± 0.0
*cpc-1 24*	12.5 ± 3.3	87.5 ± 3.3	0.0 ± 0.0	100.0 ± 0.0
*cpc-1 ttg1-23 TTG1:TTG1*	33.0 ± 1.0	67.0 ± 1.0	0.0 ± 0.0	100.0 ± 0.0
*cpc-1 ttg1-24 TTG1:TTG1*	39.7 ± 1.5	60.3 ± 1.5	0.0 ± 0.0	100.0 ± 0.0
*ttg1-1*	100.0 ± 0.0	0.0 ± 0.0	93.0 ± 1.7	7.0 ± 1.7
*ttg1-13*	100.0 ± 0.0	0.0 ± 0.0	90.7 ± 2.5	9.3 ± 2.5
*ttg1-9*	93.7 ± 0.6	6.3 ± 0.6	2.7 ± 2.5	97.3 ± 2.5
*cpc-1 ttg1-1*	56.8 ± 3.0	43.2 ± 3.0	8.5 ± 4.5	91.5 ± 4.5
*cpc-1 ttg1-13*	100.0 ± 0.0	0.0 ± 0.0	71.7 ± 2.5	28.3 ± 2.5
*cpc-1 ttg1-9*	7.0 ± 1.0	93.0 ± 1.0	0.0 ± 0.0	100.0 ± 0.0

*^1^Values indicate mean ± standard deviation of 30 roots for each genotype.*

The *ttg1-23* single mutant, separated genetically from *cpc-1*, does not exhibit an obvious defect in root epidermis development. It produces approximately 93% root-hair cells at the H-cell position and 99% non-hair cells at the N-cell position, which is comparable to the wild type (Table 1). It also exhibits a spatial distribution of *GL2:GUS* expressing cells and *GL2:GUS* non-expressing cells that is similar to the wild type (Figures 1B, 2). Overall, we conclude that the *ttg1-23* strongly enhances the reduced root-hair phenotype of *cpc-1*, but in a wild-type background, it does not significantly alter root epidermal cell-type specification.

### Identification of *TTG1* as the Mutated Gene in the Enhancer Mutant

**FIGURE 3:**
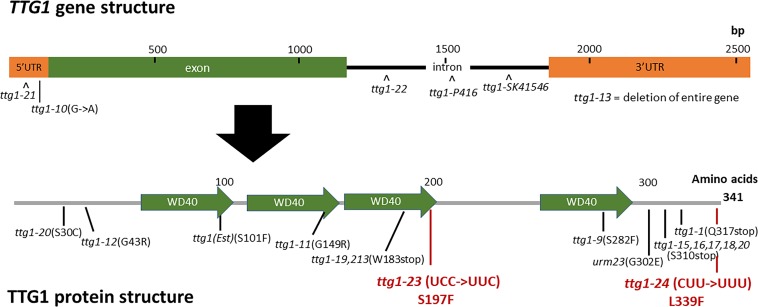
*TTG1* gene structure, protein structure, and mutations. The *TTG1* gene structure was retrieved from TAIR. The protein domains and WD40 repeats were annotated by SMART. Previously characterized mutations are indicated in black; new mutations found in this study are indicated in red.

**FIGURE 4:**
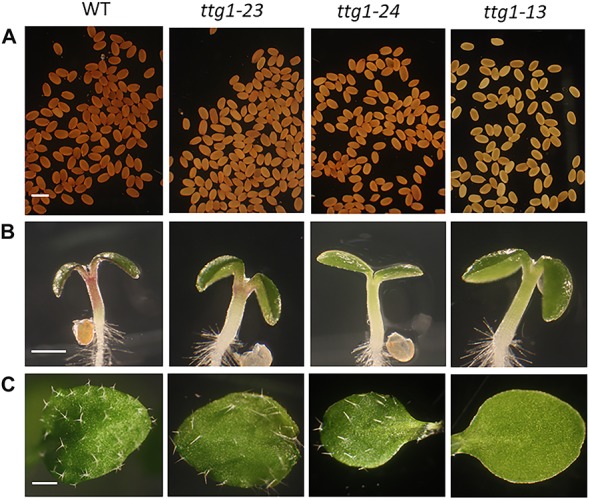
TTG1-related phenotypes in WT, *ttg1-23*, *ttg1-24*, and *ttg1-13*. **(A)** Proanthocyanidin accumulation in the seed. Bar = 1 mm. **(B)** Anthocyanin accumulation in the hypocotyl of 3-day-old seedlings. Bar = 10 mm. **(C)** Trichome production on one of the first true leaves. Bar = 2 mm.

To identify the mutated gene in the *ttg1-23* line, we conducted genetic mapping with molecular markers and whole genome sequencing (see section “Materials and Methods”). We narrowed the genetic location of the *ttg1-23* mutation to a region on chromosome 5 near the marker NGA139 at 8.4 Mb (Bell and Ecker, 1994). Upon analyzing the *ttg1-23* genome sequence in this region, we detected a single C-G to T-A substitution within the *TTG1* gene, which changes the serine encoded by the 197th codon to phenylalanine (S197F) in the predicted TTG1 protein (Figure 3). This amino acid substitution is located near the end of the third WD40 repeat (among four total WD40 repeats) of the TTG1 protein (Figure 3). Accordingly, we designated this new mutant *ttg1* allele as *ttg1-23*. Consistent with a mutation in the *TTG1* gene, the *ttg1-23* line also exhibited reduced seed coat pigmentation, reduced trichome formation, and diminished seedling anthocyanin production (Figure 4, Table 2, and Supplementary Figure S1).

**TABLE 2:** Trichome production on leaves.

Genotype	Trichome number^a^	% in clusters^b^
Wild type (WS)	45.4 ± 0.5	0.0 ± 0.0
*ttg1-1*	0.0 ± 0.0	0.0 ± 0.0
*ttg1-13*	0.0 ± 0.0	0.0 ± 0.0
*ttg1-9*	35.7 ± 0.9	22.7 ± 2.4
*ttg1-23*	28.2 ± 0.4	4.5 ± 1.1
*ttg1-24*	23.4 ± 1.4	20.1 ± 3.8
*cpc*	70.5 ± 2.5	0.3 ± 0.1
*cpc ttg1-1*	0.0 ± 0.0	0.0 ± 0.0
*cpc ttg-13*	0.0 ± 0.0	0.0 ± 0.0
*cpc ttg1-9*	40.1 ± 0.9	29.5 ± 1.3
*cpc ttg1-23*	22.6 ± 1.1	13.7 ± 0.9
*cpc ttg1-24*	9.8 ± 0.9	20.1 ± 0.3

*^*a*^Number of trichomes on the adaxial surface of one of the first true leaves (mean ± standard deviation). ^*b*^Mean percentage of trichome cells directly adjacent to another trichome cell.*

In the same EMS-based genetic screen, we identified a second *cpc-1* enhancer that resembled the *cpc-1 ttg1-23* line. This second line produced approximately 12.5% root-hair cells at the H-cell position and exhibited increased ectopic expression of *GL2:GUS* relative to *cpc-1* (Figures 1, 2, and Table 1). A line homozygous for the enhancer mutation alone, separated genetically from the *cpc-1* mutation, produced a significant root epidermal mutant phenotype, with more non-hair cells (15%) and a greater proportion of *GL2:GUS* expressing cells (16%) at the H position than wild type or *ttg1*-*23* (Figures 1, 2, and Table 1). Like the *ttg1-23* line, we found that this second enhancer mutant also exhibits reduced seed pigmentation, diminished trichome production, and reduced seedling anthocyanin (Figure 4, Table 2, and Supplementary Figure S1). Given the similar phenotypes, we sequenced the *TTG1* gene in this line and detected a single C-G to T-A substitution in codon 339, which changes a leucine to phenylalanine (L339F) near the C-terminus of the predicted 341 amino acid TTG1 protein (Figure 3). We designated the mutation in this second enhancer line as *ttg1*-*24*.

To determine whether the identified *TTG1* mutations in the *ttg1-23* and *ttg1-24* lines are responsible for their root epidermal phenotypes, we introduced a wild-type 5-kb *TTG1* genomic DNA fragment (*TTG1:TTG1* transgene, including 5’ and 3’ flanking sequences) into the *cpc-1 ttg1-23* and *cpc-1 ttg1-24* mutants. The resulting *cpc-1 ttg1-23 TTG1:TTG1* and *cpc-1 ttg1-24 TTG1:TTG1* plants each exhibit a root-hair phenotype that is comparable to the *cpc-1* single mutant (Table 1 and Supplementary Figure S2). These results indicate that the two single nucleotide changes (affecting codons 197 and 339) in the *TTG1* gene are the mutations responsible for enhancing the root epidermal phenotype of *cpc-1* in these two lines.

### Weak *ttg1* Mutant Alleles Confer a Similar Root Epidermal Phenotype

Our finding that the *ttg1-23* and *ttg1-24* mutations enhance the *cpc-1* phenotype (i.e., increase the production of non-hair cells) was unexpected, because previous studies have shown that *ttg1* mutants produce more root-hair cells and thus *TTG1* is likely involved in non-hair cell specification (Galway et al., 1994). To investigate this discrepancy, we examined additional mutant alleles of the *TTG1* gene which have not been analyzed in detail for their root-hair phenotypes (Zhang and Schrader, 2017). We selected three previously described *ttg1* mutant lines: *ttg1-1*, *ttg1-9*, and *ttg1-13*. The *ttg1-1* bears a single C-to-T mutation that introduces a stop codon in place of a glutamine codon at position 317 (Figure 3), leading to a truncated TTG1 protein lacking the C-terminal 25 amino acid residues (Walker et al., 1999). The *ttg1-13* mutation is a large deletion (>4 kb) encompassing the entire *TTG1* gene (Larkin et al., 1999). Consistent with the nature of their mutations, both *ttg1-1* and *ttg1-13* are known to exhibit strong (presumed null) mutant phenotypes for other TTG1-related processes. In contrast, the *ttg1-9* mutant is reported to exhibit relatively weak TTG1-related phenotypes, and it contains a C-to-T substitution causing an amino acid change from serine to phenylalanine at position 282 (S282F) (Figure 3; Larkin et al., 1994b; Walker et al., 1999).

**FIGURE 5:**
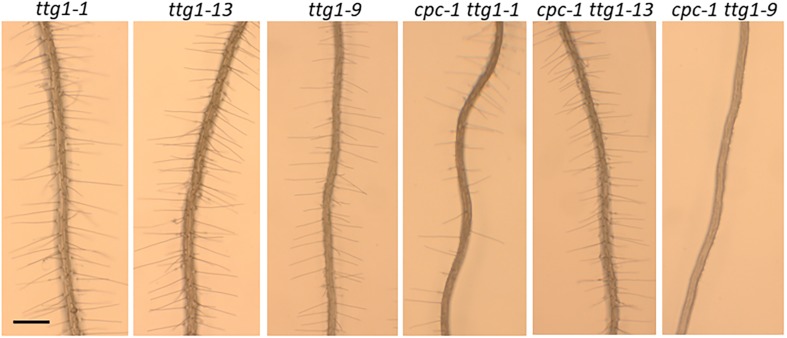
Seedling roots of *ttg1-1*, *ttg1-13*, *ttg1-9*, *cpc-1 ttg1-1*, *cpc-1 ttg1-13*, and *cpc-1 ttg1-9* displaying their root hair phenotypes. Bar = 200 μm.

To compare the effects of these three *ttg1* mutations (*ttg1-1*, *ttg1-9*, and *ttg1-13*) with *ttg1-24*, *ttg1-23*, and wild-type, we quantified the root epidermis cell types produced in each single mutant and each *cpc-1* double mutant. We found that the *ttg1-1* and *ttg1-13* roots exhibit a strong hairy phenotype, with root-hair cells produced throughout the H position (100% hair cells) and in most of the N position (91 and 93% hair cells) (Table 1 and Figure 5). In contrast, the *ttg1-9* roots exhibit a minor cell-type defect, with only a small fraction of ectopic hair cells in the N-position (3%) and ectopic non-hair cells in the H position (6%) (Table 1 and Figure 5). This *ttg1-9* phenotype is comparable to the mild root epidermal phenotypes exhibited by the *ttg1-23* and *ttg1-24* mutants (Table 1). These results suggest that strong defects in TTG1 function (e.g., truncated protein or gene deletion) essentially prevent non-hair cell specification, whereas weak TTG1 mutations (e.g., missense mutations) have a mild effect on root epidermal cell specification.

The analysis of double mutants with *cpc-1* showed a similar trend. The *cpc-1 ttg1-1* double mutant produced 57% root-hair cells at the H position and 9% root-hair cells at N position, and *cpc-1 ttg1-13* produced 100% root-hair cells at the H position and 72% root-hair cells at the N position (Table 1 and Figure 5). This indicates that these two strong *ttg1* mutations do not enhance *cpc-1* but instead, they suppress the *cpc-1* mutant phenotype. Interestingly, the *cpc-1 ttg1-9* double mutant exhibits a nearly hairless root phenotype, similar to *cpc-1 ttg1-23* and *cpc-1 ttg1-24* (Table 1 and Figure 5), indicating that *ttg1-9* enhances the *cpc-1* mutant phenotype.

We also examined the relative effect of these *ttg* mutants and *cpc ttg* double mutants on trichome formation. Consistent with the root epidermal phenotypes, we observed two distinct classes of trichome mutant phenotypes. The *ttg1-1* and *ttg1-13* produce glabrous leaves, and inclusion of *cpc-1* into these mutants (i.e., *cpc-1 ttg1-1* and *cpc-1 ttg1-13*) does not alter this phenotype (Table 2). On the other hand, *ttg1-9, ttg1-23*, and *ttg1-24* are able to produce a substantial number of leaf trichomes (approximately 50–80% of the wild-type number), with a fraction of these present in clusters (Table 2). The *cpc-1 ttg1-9*, *cpc-1 ttg1-23*, and *cpc-1 ttg1-24* double mutants produce a similar number of trichomes and fraction of trichome clusters, but significantly less than the *cpc-1* mutant (Table 2).

These results show that the *ttg1-9*, *ttg1-23*, and *ttg1-24* mutations generate similar effects on epidermal cell differentiation, which are distinct from the effects of the strong *ttg1-1* and *ttg1-13* mutations. Given that *ttg1-9*, *ttg1-23*, and *ttg1-24* alter different residues of the TTG1 protein, it is likely that their common phenotypes represent the general impact of a partially functional TTG1 protein.

### The *ttg1* Mutations Differentially Affect Target Gene Expression

Next, we sought to understand how the various *ttg1* mutants yield their distinct effects on root epidermal patterning and enhancement of the *cpc-1* phenotype. As a component of the central MBW complex that specifies root epidermal cell fates, TTG1 is believed to participate in transcriptional activation of multiple target genes. These include the presumed direct activation of four target genes: *GL2*, *MYB23*, *CPC*, and *TRY* genes (Schiefelbein et al., 2014). We analyzed the transcript level for these four genes in the developing root from the five *ttg1* mutants and wild type using quantitative real-time PCR.

**FIGURE 6:**
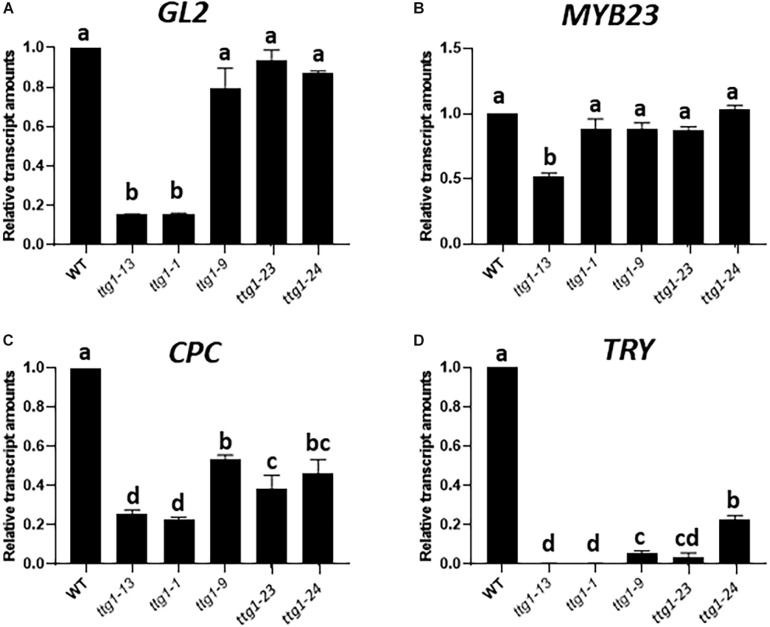
Relative transcript amounts from **(A)** *GL2*, **(B)** *MYB23*, **(C)** *CPC*, and **(D)** *TRY* genes from seedling root tips of WT, *ttg1-13, ttg1-1, ttg1-9, ttg1-23*, and *ttg1-24*, determined with RT-qPCR. Three independent biological replicates were performed for each genotype. Error bars represent standard deviations. For each panel, bars marked with the same letter indicate values not significantly different; bars marked with different letters indicate values showing statistically significant differences (*P* < 0.05; ANOVA testt).

The most striking result from this analysis is the different effects on *GL2* expression. The *GL2* transcript level is dramatically reduced in the strong *ttg1-1* and *ttg1-13* mutants, whereas the weak *ttg1-9*, *ttg1-23* and *ttg1-24* show near-normal *GL2* levels (Figure 6A). Given the critical role of GL2 in specifying the non-hair cell type, these differences in *GL2* expression among the *ttg1* mutants are consistent with the root epidermal phenotypes of the corresponding mutants. Specifically, the *ttg1-1* and *ttg1-13* mutants essentially lack non-hair cells (Table 1) and *ttg1-1* produces a very low level of *GL2:GUS* transcriptional reporter expression (Hung et al., 1998). On the other hand, the *ttg1-9*, *ttg1-23*, and *ttg1-24* produce near-normal amounts of non-hair cells (Table 1), and *ttg1-23* and *ttg1-24* generate near-normal *GL2:GUS* transcriptional reporter expression in the developing root epidermis (Figure 1). The strong correlation between *GL2* gene expression and epidermal phenotypes suggest that differences in the ability of the various *ttg1* mutant proteins to activate *GL2* expression is primarily responsible for their different root epidermis phenotypes.

The transcript levels of *CPC* and *TRY*, the two genes encoding lateral inhibitors, are decreased in each of the *ttg1* mutants (Figures 6C,D). Interestingly, the weak *ttg1* mutants (*ttg1-9*, *ttg1-23*, and *ttg1-24*), possess a higher level of *CPC* RNA than the strong *ttg1* mutants (*ttg1-1*, *ttg1-13*), which may enable the weak *ttg1* mutants to achieve lateral inhibition and explain why they exhibit a near-normal root-hair pattern. Indeed, the effect of *cpc-1* on the root epidermis phenotype of these weak *ttg1* mutants (Table 1) demonstrates that they possess CPC function because the root-hair production and pattern formation in these weak *ttg1* mutants is CPC-dependent. It is notable that *TRY* transcript levels are strongly diminished, relative to *CPC*, in all *ttg1* mutants (Figure 6D). The relatively low level of *TRY* expression compared to *CPC* in the weak *ttg1* mutants may provide an explanation for the ability of the weak *ttg1* mutants to enhance the *cpc-1* phenotype (see section “Discussion”). Finally, we observe substantial *MYB23* transcript accumulation in the *ttg1* mutants and wild type, indicating that TTG1 is perhaps not essential for transcription of *MYB23* (Figure 6B).

### Mutant TTG1 Proteins Exhibit Altered Interaction With GL3

**FIGURE 7:**
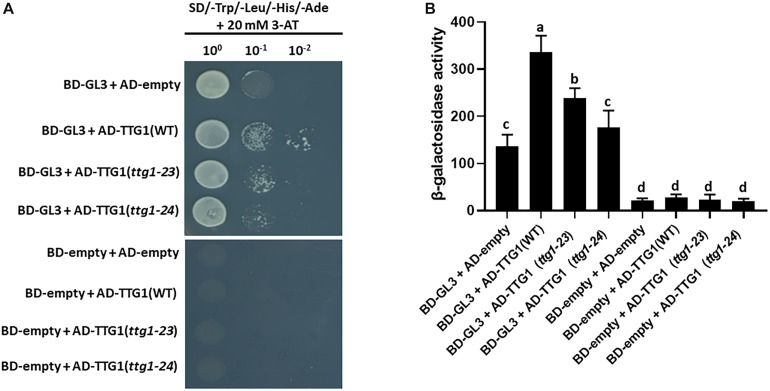
Yeast two-hybrid protein interaction assays. **(A)** The growth of yeast cells harboring the GL3 fused to the GAL4 DNA-binding domain (BD-GL3) or the GAL4 DNA-binding domain alons (BD-empty), and TTG1(WT), TTG1(*ttg1-23*), or TTG1(*ttg1-24*) fused to the GAL4 activation domain (AD) are shown. The yeast cells are grown on the SD/-Trp/-Leu/-His/-Ade selection medium with 20 mM 3-aminotriazole (3-AT). For serial dilution analysis, yeast cells were collected and adjusted to OD_600_ = 1.0, and diluted to 10^0^, 10^–1^, and 10^–2^. **(B)** Yeast two-hybrid β-galactosidase activity assay quantifying the interaction between GL3 and TTG1(WT), TTG1(*ttg1-23*) or TTG1(*ttg1-24*). Three independent biological replicates were performed for each combination. Error bars represent standard deviations from the three replicates. Bars marked with the same letter indicate values not significantly different; bars marked with different letters indicate values showing statistically significant differences (*P* < 0.05; ANOVA test).

In the MBW complex for root epidermal patterning, TTG1 is reported to directly interact with the GL3 bHLH protein (Payne et al., 2000). To determine whether the mutated TTG1 proteins in *ttg1-23* and *ttg1-24* have altered interaction with GL3, we conducted a yeast two-hybrid assay. The GL3 cDNA was fused to the sequence encoding the GAL4 DNA binding domain (BD), and three different TTG1 cDNAs (representing wild type *TTG1*, *ttg1-23*, and *ttg1-24* gene products) were fused to sequences encoding the GAL4 activation domain (AD). The introduction of BD-GL3 and AD-empty constructs into yeast cells yielded some *lacZ* reporter expression, but higher reporter expression was achieved in yeast co-expressing BD-GL3 fusion together with the AD-TTG1 fusion (Figure 7). Significantly, lower β-galactosidase activities were detected in yeast cells co-expressing BD-GL3 fusions with AD-TTG1(*ttg1-23*) and AD-TTG1(*ttg1-24*) than those co-expressing BD-GL3 with AD-TTG1(WT) (Figure 7). As controls, yeast cells co-expressing BD-empty together with AD-empty, AD-TTG1(WT), AD-TTG1(*ttg1-23*), or AD-TTG1(*ttg1-24*) were not able to grow on the selection plate, and very low β-galactosidase activities were measured in these combinations (Figure 7). These results indicate that all three tested TTG1 proteins are able to physically interact with the GL3 bHLH protein in yeast, but the mutated TTG1 proteins (from *ttg1-23* and *ttg1-24*) have a diminished ability to interact. This suggests that the amino acid changes in *ttg1-23* and *ttg1-24* alter protein-protein binding of TTG with GL3, which may reduce the effectiveness of the MBW complex and lead to the observed root epidermis defects.

### Accumulation of Wild-Type and Mutated TTG1 Proteins in the Root Epidermis

**FIGURE 8:**
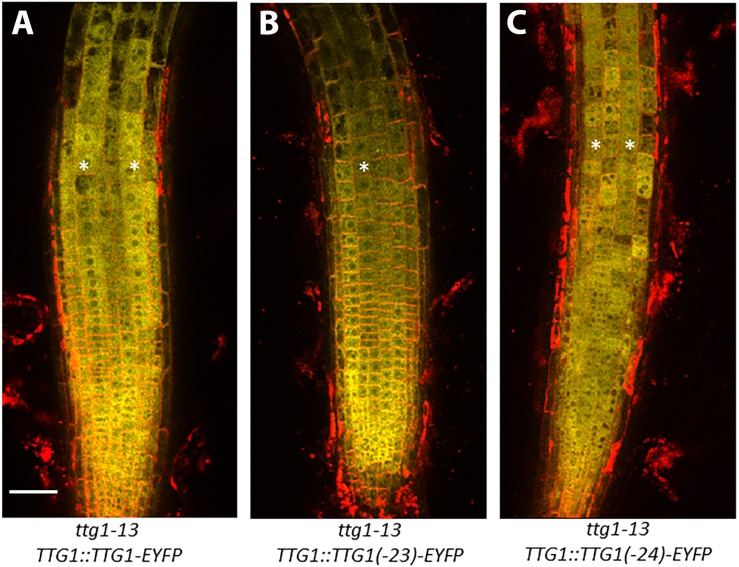
Accumulation of TTG1-EYFP fusion proteins in *ttg1-13* seedling roots bearing the **(A)** *TTG1:TTG1*(WT)*-EYFP*, **(B)** *TTG1:TTG1*(*ttg1-23*)*-EYFP*, or **(C)** *TTG1:TTG1*(*ttg1-24*)*-EYFP* transgene. White stars mark the H-position epidermal cell files. The red color represents propidium iodide and the yellow color represents YFP. Representative images are shown from the analysis of at least 3 independent single-insertion lines for each transgenic experiment. Bar = 50 μm.

To determine whether the *ttg1-23* and *ttg1-24* mutations alter the accumulation of TTG1 protein in the root epidermal cells, we generated *EYFP* translational fusions to three versions of *TTG1* (from wild type, *ttg1-23*, and *ttg1-24*) in the *ttg1-13* mutant background (which bears a deletion of TTG1). Transgenic plants bearing the wild-type *TTG1:TTG1-EYFP* construct showed TTG1-EYFP accumulation in all epidermal cells (both H- and N-position) and predominantly in the cytoplasm (Figure 8). Both the *TTG1:ttg1-23-EYFP* and *TTG1:ttg1-24-EYFP* proteins exhibit a similar EYFP accumulation pattern as *TTG1-EYFP* (Figure 8), indicating that the *ttg1-23* and *ttg1-24* mutations have little if any effect on the cellular accumulation of the TTG1 protein.

## Discussion

The TTG1 protein has been known to be a component of the MBW complex that regulates root-hair patterning in Arabidopsis (Galway et al., 1994). However, the precise role and importance of TTG1 for the function of the MBW complex has not been defined. Previously reported strong *ttg1* mutants were found to produce all root-hair cells in their root epidermis, suggesting that TTG1 is involved in specifying the non-hair cell type (Galway et al., 1994). In another study, ectopic overexpression of the bHLH factors *GL3* and/or *EGL3* in the *ttg1* mutant background yielded an entirely hairless root (Bernhardt et al., 2003), implying that TTG1 is not essential for the MYB-bHLH induction of non-hair gene transcription but may influence its level or activity. Here, we surprisingly found that two new *ttg1* mutants cause an increase in non-hair cells and enhance the *cpc-1* mutation, which is distinct from the hairy root phenotype of the strong *ttg1-1* and *ttg1-13* mutants. Our analysis of target gene expression in these lines provides an explanation for these different *ttg1* mutant phenotypes. Although strong *ttg1* mutants have very low levels of *GL2*, *CPC*, and *TRY* transcripts, our new *ttg1* mutants exhibit differences in the expression of the various MBW target genes. *GL2* gene expression is not significantly reduced, yet *CPC* and *TRY* gene expression is substantially affected. This means that, in our new *ttg1* mutants, the non-hair cell fate will tend to be induced to a greater extent than normal, which is consistent with the observed phenotypes. Taken together, our study suggests that TTG1 is best considered as a component of the MBW complex that is necessary to ensure the proper relative activation of downstream target genes to generate the appropriate proportion and pattern of the root-hair and non-hair cell types.

In addition to the two new *ttg1* mutants identified in this study (*ttg1-23* and *ttg1-24*), we also discovered that a previously isolated *ttg1* missense mutant (*ttg1-9*) produced a similar effect on root epidermis development. Thus, we found that three independent amino-acid substitution variants of the TTG1 protein generate essentially the same phenotype, including a mild defect in root epidermal pattern, an ability to enhance the *cpc-1* reduced-hair phenotype, and a distortion in the relative expression of MBW target genes. The locations of the amino acid substitutions in these three mutants are dispersed along the TTG1 protein (residues 197, 282, and 339), suggesting that their common phenotype is not due to alteration of a particular protein motif or domain. Our yeast two-hybrid analysis of the ttg1-23 and ttg1-24 proteins showed a reduction in their ability to interact with their partner bHLH (GL3) protein (Figure 7). Also, we observed a comparable accumulation of EYFP-tagged proteins for the ttg1-23 and ttg1-24 variants as for the wild-type TTG1 (Figure 8). Together, these findings suggest that the similar phenotype of these *ttg1* missense mutants is the result of a common negative effect of these amino acid substitutions on TTG1-bHLH interaction, which leads to reduced MBW complex formation and altered target gene expression. This explanation implies that multiple residues/regions of the TTG1 protein are likely to be important for proper TTG1-bHLH interaction. In a previous study, the C-terminal 25 amino acids of the TTG1 protein were shown to be essential for interaction with GL3 in yeast (Payne et al., 2000). Our results also imply that the MBW target genes possess differential sensitivity to the level of the MBW complex. In particular, among the four target genes tested, expression of the *TRY* gene appears to be most sensitive to MBW level, whereas *GL2* is least sensitive (Figure 6). In this respect, it is notable that the requirement for TTG1 (i.e., the requirement for a MBW complex) for non-hair cell differentiation can be bypassed by overexpression of GL3 or EGL3 (Bernhardt et al., 2003), suggesting that high levels of the bHLH and MYB components are sufficient to induce target gene expression. In future studies, it will be informative to analyze the presumed differential protein-DNA interactions in greater detail to evaluate the importance of MBW complex on its various downstream targets.

The ability of the *ttg1-23* and *ttg1-24* mutations to enhance, rather than suppress, the *cpc-1* mutant phenotype was initially puzzling. However, given the effect of these mutations on MBW target gene expression, we can provide the following explanation. First, it is known that *TRY* encodes a CPC-like R3-type MYB protein, which is partially functionally redundant with *CPC*, and mutations in *TRY* are known to enhance the *cpc-1* phenotype (Simon et al., 2007). We showed that each of the weak *ttg1* mutants are defective in *TRY* gene expression (Figure 6). Therefore, in the *cpc-1* mutant background, these weak *ttg1* mutants will essentially lack the ability to produce the CPC/TRY lateral inhibitory proteins, due to the combination of the *cpc* mutation and their inability to activate *TRY* transcription. However, these weak *ttg1* mutants retain substantial capacity to activate *GL2* and *MYB23* gene expression, which promote the non-hair cell fate. Thus, with respect to the root epidermal patterning process, these *cpc-1 ttg1* mutants essentially behave like a *cpc try* mutant and, accordingly, result in a hairless root phenotype.

From an evolutionary point of view, it is interesting that single amino acid substitutions in the TTG1 protein are able to modify the pattern of epidermal cell types in the Arabidopsis root. In particular, the *ttg1-24* mutant exhibits a novel distribution of root-hair cells and non-hair cells, significantly different from the wild-type (Table 1). This suggests that TTG1 could provide a target for mutations that can yield novel and potentially beneficial distributions of root-hair cells and non-hair cells in plants. It is notable that none of the sequenced natural accessions of *Arabidopsis thaliana* exhibit differences in their predicted *TTG1* protein product^1^. The future molecular characterization of root hair patterns in a broad array of plant species will likely uncover the mechanisms responsible for the evolution of new cell type patterns in plant roots.

## Data Availability Statement

The datasets used in this study can be found in the NCBI using accession number PRJNA610815.

## Author Contributions

YL conducted the experiments. Both authors analyzed the data, designed the experiments, wrote the manuscript, and approved the final version of the manuscript.

## Conflict of Interest

The authors declare that the research was conducted in the absence of any commercial or financial relationships that could be construed as a potential conflict of interest.

## Footnotes

1 www.1001genomes.org
