# Supplementary material for: Novel TTG1 Mutants Modify Root-Hair Pattern Formation in Arabidopsis
Source: Front Plant Sci. 2020 Apr 7;11:383. doi: 10.3389/fpls.2020.00383 (PMC7154166; doi:10.3389/fpls.2020.00383)
Supplement: Supplementary file 1 [file Data_Sheet_1.PDF]

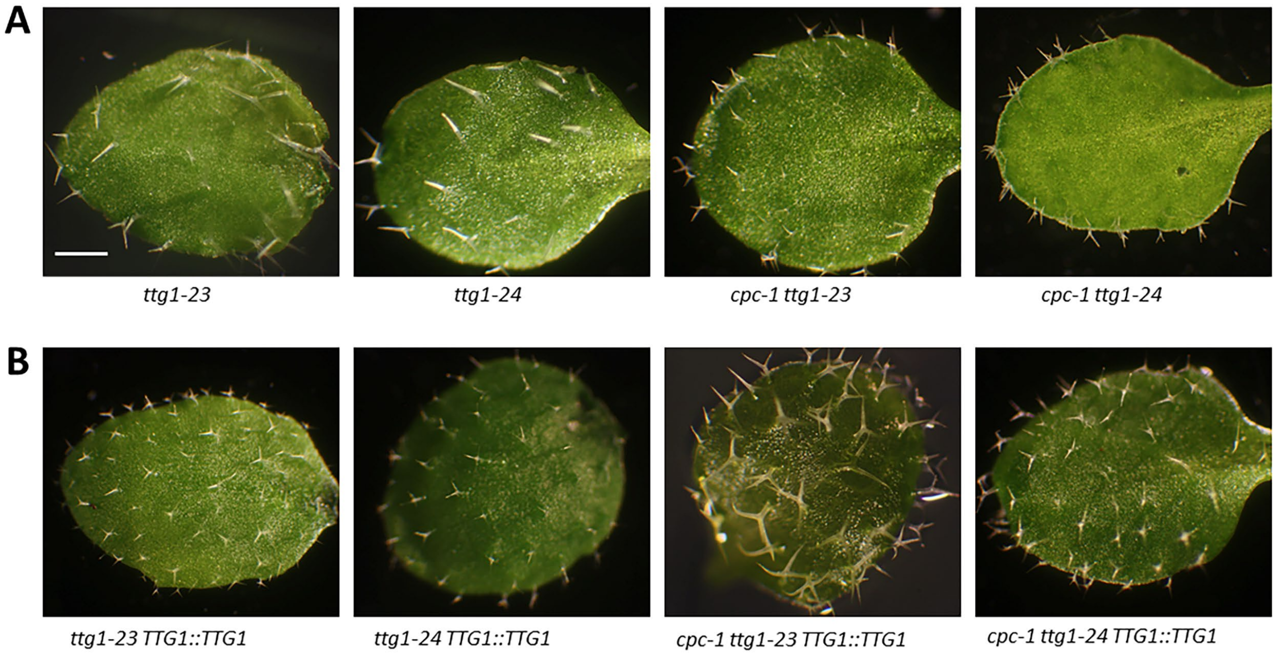

Supplemental Figure S1. Trichome phenotypes of *ttg1-23*, *ttg1-24*, *cpc-1 ttg1-23*, *cpc-1 ttg1-24*, *ttg1-23 TTG1::TTG1*, *ttg1-24 TTG1::TTG1*, *cpc-1 ttg1-23 TTG1::TTG1*, and *cpc-1 ttg1-24 TTG1::TTG1*. One of the first true leaves is displayed in each panel. Bar = 2 mm.
