# Supplementary material for: Novel TTG1 Mutants Modify Root-Hair Pattern Formation in Arabidopsis
Source: Front Plant Sci. 2020 Apr 7;11:383. doi: 10.3389/fpls.2020.00383 (PMC7154166; doi:10.3389/fpls.2020.00383)
Supplement: Supplementary file 3 [file Table_1.DOCX]

**Supplemental Table S1.** List of primer sequences used in this research.

| **Function** | **Name** | **Sequence** |
| --- | --- | --- |
| *ttg1-23* genotyping | dCAPS_ar77_ScrFI_F | CCTTATCACGTAAATCAAAGATCCTCCCG |
|  | dCAPS_ar77_ScrFI_R | CTTGTAGTATTGATACGACGTGTACG |
| *ttg1-24* genotyping | dCAPS_ar14_ScrFI_F | CGAAACAGAAACTCTCACCTCAAACTCCAG |
|  | dCAPS_ar14_ScrFI_R | TCCGATGTCGGTTTATTCGGCTG |
| *TTG1::TTG1* cloning | primer1 | ACCGCGGTGGCGGCCGCTATTCATACATATCCAAGAGAC |
|  | primer2 | CGTAGCTTCTATCTCCTTGTTCACGAATAGAG |
|  | primer3 | CTCTATTCGTGAACAAGGAGATAGAAGCTACG |
|  | primer4 | AATTCCTGCAGCCCGGGGGAATTCTACTATTAATGGTT |
| *TTG1::TTG1-EFYP* cloning | primer5 | ACCGCGGTGGCGGCCGCTATTCATACATATCCAAGAGAC |
|  | primer6 | CGTAGCTTCTATCTCCTTGTTCACGAATAGAG |
|  | primer7 | CTCTATTCGTGAACAAGGAGATAGAAGCTACG |
|  | primer8 | AATTCCTGCAGCCCGGGGGAATTCTACTATTAATGGTT |
|  | primer9 | CCTCGCCCTTGCTCACCATAACTCTAAGGAGCTGCATTTTG |
|  | primer10 | CAAAATGCAGCTCCTTAGAGTTATGGTGAGCAAGGGCGAGG |
|  | primer11 | GAAACAGAAACTCTCACCTCACTTGTACAGCTCGTCCATG |
|  | primer12 | CATGGACGAGCTGTACAAGTGAGGTGAGAGTTTCTGTTTC |
| RT-qPCR | qRT-CPC-F | ATAAACTCGTTGGCGACAGGTG |
|  | qRT-CPC-R | AACGACGCCGTGTTTCATAAGC |
|  | qRT-TRY-F | GTACAGACTTGTCGGTGATAGGTG |
|  | qRT-TRY-R | GAAGCTGGCGTCGTTTATCAGC |
|  | qRT-ETC1-F | GCACGGTGCGATCGTAAATCTTTG |
|  | qRT-ETC1-R | CCACTCAAGACTGCTCACTTCTTC |
|  | qRT-GL2-F | GCAACTCAGTGGCAATCCAGAC |
|  | qRT-GL2-R | TGTCTTGCAGCACCCATATGCTC |
|  | qRT-GL3-F | ACCGTCAATTGCAAGCACAAGG |
|  | qRT-GL3-R | GCAACCCTTTGAAGTGCTTCTTTG |
|  | qRT-EGL3-F | AAGTGCTTCGCTTCAGACAGTG |
|  | qRT-EGL3-R | TTCGGTCGTGCCAATCTCAAGG |
|  | qRT-WER-F | AAGAGGCAATTTCACCGAGCAAG |
|  | qRT-WER-R | CCAAGCAACTTGTGGAGCCTAATG |
|  | qRT-MYB23-F | ACAAGCTCCTCGGCAACAGATG |
|  |  |  |
