# Supplementary material for: Novel TTG1 Mutants Modify Root-Hair Pattern Formation in Arabidopsis
Source: Front Plant Sci. 2020 Apr 7;11:383. doi: 10.3389/fpls.2020.00383 (PMC7154166; doi:10.3389/fpls.2020.00383)
Supplement: Supplementary file 2 [file Data_Sheet_2.PDF]

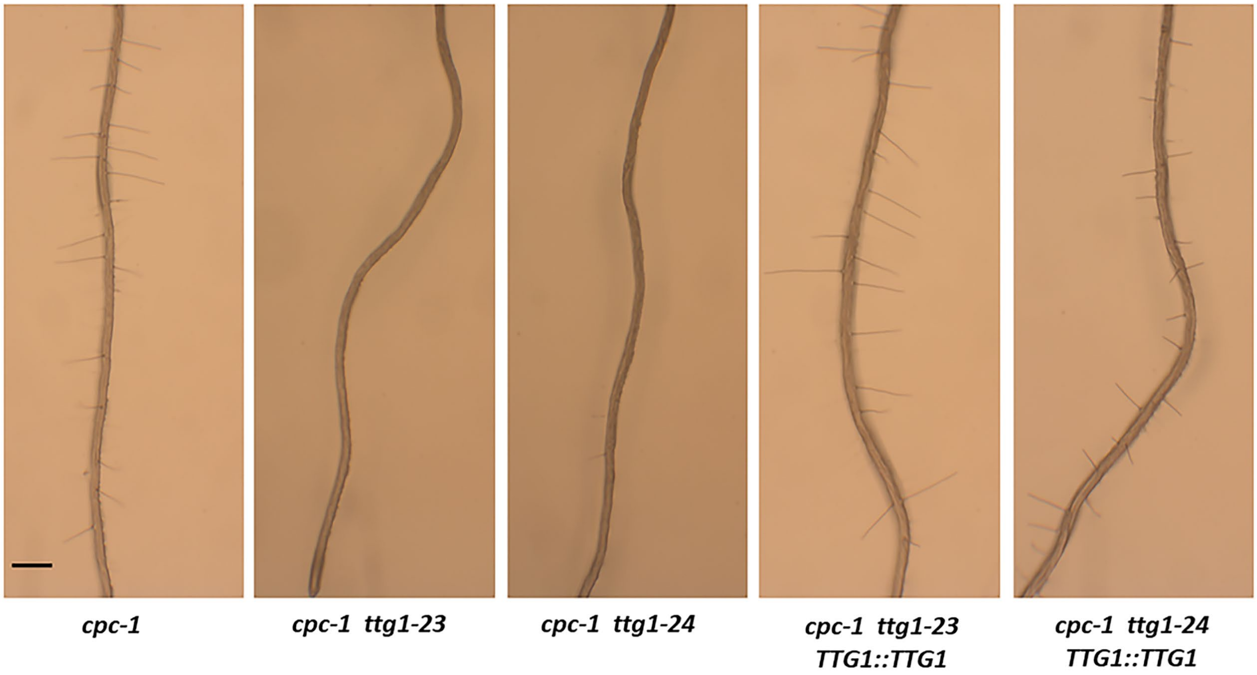

Supplemental Figure S2. Seedling roots of *cpc-1*, *cpc-1 ttg1-23*, *cpc-1 ttg1-24*, *cpc-1 ttg1-23 TTG1::TTG1*, and *cpc-1 ttg1-24 TTG1::TTG1*. Bar = 0.2 mm.
